# Pazopanib in Patients with Osteosarcoma Metastatic to the Lung: Phase 2 Study Results and the Lessons for Tumor Measurement

**DOI:** 10.1155/2022/3691025

**Published:** 2022-01-15

**Authors:** Paul Frankel, Chris Ruel, An Uche, Edwin Choy, Scott Okuno, Neeta Somiah, Warren A. Chow

**Affiliations:** ^1^Division of Biostatistics, Department of Computational and Quantitative Medicine, City of Hope National Medical Center, Duarte, CA, USA; ^2^Department of Hematology & Oncology, Highland Hospital, Oakland, CA, USA; ^3^Department of Hematology/Oncology, Massachusetts General Hospital, Boston, MA, USA; ^4^Division of Medical Oncology, Department of Oncology, Mayo Clinic, Rochester, MN, USA; ^5^Department of Sarcoma Medical Oncology, MD Anderson Cancer Center, Houston, TX, USA; ^6^Division of Hematology/Oncology, Department of Medicine, UCI Health, Orange, CA, USA

## Abstract

**Background:**

This single-arm, multicenter, phase 2 study evaluated the safety and antitumor activity of pazopanib in patients with unresectable, pulmonary metastatic osteosarcoma. *Patients and Methods*. Patients with pulmonary metastatic osteosarcoma unresponsive to chemotherapy were eligible. Patients who received prior tyrosine kinase inhibitor therapy were excluded. Pazopanib at 800 mg once daily was administered for 28-day cycles. Tumor responses were evaluated by local radiology assessment 1 month prior to and after initiation of treatment to calculate tumor doubling time and after every even numbered cycle. The primary endpoints were progression-free survival at 4 months, concomitant with a demonstrated 30% increase in tumor doubling time relative to the pretreatment growth rate.

**Results:**

12 patients (7 female) were enrolled. The study was terminated prematurely due to withdrawal of financial support by the sponsor. 8 subjects were eligible for the primary analysis, whereas 4 patients were in a predefined exploratory “slow-growing” cohort. In the “fast-growing” cohort, 3 of the 8 patients (37.5%) eligible for first-stage analysis were deemed “success” by the preplanned criteria, adequate to proceed to second-stage accrual. In addition, 1 of the 4 patients in the “slow-growing” cohort experienced a partial remission. Grade 1-2 diarrhea was the most common adverse event, and grade 3 events were infrequent.

**Conclusion:**

This study illustrates a novel method of demonstrating positive drug activity in osteosarcoma by increasing tumor doubling time, and this is further supported by a partial response in a patient with “slow-growing” disease. This trial is registered with NCT01759303.

## 1. Introduction

Osteosarcoma is the most common malignant primary bone tumor [1]. It is characterized by abnormal osteoid production associated with malignant mesenchymal cells [2]. The U.S. incidence of osteosarcoma is 1,000 cases/year [3]. Osteosarcoma follows a bimodal distribution, with an initial peak in adolescence and a second after the 6th decade [4]. Prior to acceptance of perioperative chemotherapy, 5-year overall survival (OS) was <20% despite optimal surgery [4]. This has significantly improved; the recent European-American (EURAMOS) phase 3 trial investigated intensified postoperative chemotherapy for patients <40 years with nonmetastatic osteosarcoma of the extremity whose tumor showed poor response to preoperative chemotherapy (≥10% viable tumor). The estimated 3-year OS rate was >70% [5].

Unfortunately, a significant number of patients with nonmetastatic osteosarcoma still relapse, with the lungs being the most common and often only site of distant spread. This is a consequence of dissemination almost exclusively through the vascular system as the lungs possess a rich vascular supply [6]. Patients with osteosarcoma who develop lung metastases generally have a poor prognosis with the exception of those with a limited number of metastases and prolonged disease-free interval (DFI) [7, 8]. However, recent analysis of the 5-year OS of patients who develop pulmonary metastases and are treated with metastasectomy with or without chemotherapy is only 30–39% [7, 8]. Patients who present with synchronous pulmonary metastases at diagnosis (10–20% of patients with osteosarcoma) and treated with chemotherapy have even poorer prognosis (5-year OS 29%) [9]. Thus, novel therapies are needed for this population.

Preclinical studies have shown that the vascular endothelial growth factor (VEGF) pathway is a critical signaling pathway in osteosarcoma, and VEGF receptor (VEGF-R) expression was correlated with an increased incidence of pulmonary metastasis and decreased event-free survival and OS [10, 11]. Accordingly, targeting VEGF-R is appealing.

Pazopanib is an oral tyrosine kinase inhibitor of VEGF-R-1,2,3, platelet-derived growth factor receptor-*α*,*β* (PDGFR-*α*,*β*), and c-Kit [12]. Pazopanib is effective and thus widely used in patients with metastatic soft-tissue sarcoma who have failed prior therapy; however, its efficacy in bone sarcomas has not been extensively studied [13]. Responses in bone sarcomas have been reported in phase 1 studies of pazopanib. In a phase 1 trial of pazopanib, two patients with chondrosarcoma experienced stable disease lasting 7.6 and 19.8 months [14]. Additionally, in a Children's Oncology Group (COG) phase 1 study of pazopanib in children with refractory solid tumors, 1/1 subjects with osteosarcoma maintained stable disease (SD) >6 cycles [15]. These anecdotal reports of prolonged SD in chondrosarcoma and in osteosarcoma provided the rationale for this multiinstitutional, phase 2 trial of pazopanib in patients with pulmonary metastatic osteosarcoma.

## 2. Materials and Methods

### 2.1. Patients

Patients aged ≥16 years with osteosarcoma with surgically unresectable lung metastases (imaging reviewed by experienced oncologic thoracic surgical oncologists) who progressed or relapsed on prior chemotherapy were eligible. Patients must have had ≥1 multiagent chemotherapy either in the neoadjuvant or adjuvant settings. Patients were allowed to have received 0–2 lines of therapy for metastatic disease. Other inclusion criteria were as follows: Eastern Cooperative Oncology Group (ECOG) performance status of 0–2, measurable disease as outlined in RECIST version 1.1, and adequate cardiac, hepatic, renal, and hematopoietic function. Women of childbearing potential agreed to use acceptable contraception. Exclusion criteria included the following: prior treatment with any other anticancer therapy within 14 days (or 5 half-lives, whichever is longer) of the 4-week drug holiday preceding study treatment, prior malignancy, except if disease-free for 3 years or history of resected nonmelanomatous skin carcinoma, evidence of CNS involvement, except if previously treated, currently asymptomatic, and not requiring treatment, and other clinically significant conditions that affected the safety of the patient or was expected to interfere with the assessment of study variables.

### 2.2. Treatment

Patients ≥18 years self-administered pazopanib 800 mg once daily on a continuous 28-day cycle until disease progression or unacceptable toxicity. Patients between 16 and 18 years and with BSA <1.6 m^2^ received pazopanib 600 mg once daily on the same schedule, but all enrolled patients were ≥18 years. Guidelines for pazopanib dose interruption or modification followed standard of care in the event of treatment-emergent adverse events (TEAEs). Generally, pazopanib was withheld for any ≥grade 2 event and resumed when adverse event (AE) improved to ≤grade 1 or baseline with dose reduced by 200 mg daily. The minimum pazopanib dose allowed was 400 mg daily.

### 2.3. Safety Assessments

Baseline assessments were performed within 28 days prior to the start of study treatment and included detailed medical and medication history, physical examination (including weight, height, and BSA for patients <18 years of age), blood pressure, ECOG performance status, pregnancy testing (if applicable), complete blood count, 24-hour urine collection for creatinine and protein, comprehensive metabolic panel, coagulation parameters, testing of thyroid stimulating hormone and lipase enzyme, 12-lead electrocardiogram, and echocardiography or multigated acquisition (MUGA) scan. These assessments were scheduled and repeated regularly throughout the study.

### 2.4. Efficacy Assessments

Computed tomography (CT) of the chest was performed 4 weeks prior to start of treatment and 3–5 calendar days prior to the start of the study in order to calculate baseline tumor growth rate. Chest CT was repeated at completion of cycle 1, cycle 2, cycle 4, and then after every 2 cycles, thereafter, until the end of treatment. All other scans (CT abdomen/pelvis, PET/CT, or MRI) were performed as clinically indicated.

Tumor response was assessed using RECIST version 1.1 guidelines at each imaging time point. Bidimensional tumor measurements (i.e., longest dimension of the tumor(s) and its longest perpendicular dimension) at each imaging time point were recorded and sent to Vector Oncology (clinical research organization) within 2 working days of each imaging assessment to calculate tumor growth rate.

If the criteria for completion response (CR) or partial response (PR) were met, repeat measurements within 4 weeks were performed for confirmation. Subjects were allowed to continue study treatment in the absence of disease progression or unacceptable toxicity. AEs were recorded and graded using the Common Terminology Criteria for Adverse Events (CTCAE) version 4.0.

### 2.5. Pharmacokinetic (PK) Analysis

Pazopanib trough steady-state concentration was measured on day 1 of cycle 2 prior to its administration. Plasma samples were analyzed using a validated high-performance liquid chromatography tandem mass spectrometry method [14]. Pazopanib was extracted from 50 *μ*L of human plasma by protein precipitation. Extracts were analyzed by high-performance liquid chromatography tandem mass spectrometry using a Turbo-IonSpray interface and positive-ion, multiple reaction monitoring. The lower limit of quantitation for pazopanib was 10.0 mg/L [14].

### 2.6. Statistical Analysis

This was a prospective, single-arm, open-label, multisite phase 2 trial. The primary endpoints were progression-free survival (PFS) at 4 months, concomitant with a demonstrated 30% increase in tumor doubling time (DT) relative to the pretreatment growth rate. Tumor growth rates were estimated based on serial measurements of pulmonary lesions. For subjects who had multiple lung lesions, each tumor was followed independently. The median tumor growth rate (equivalent to the tumor DT) was used as the subject's tumor growth rate. The primary measure of growth was based on the single longest dimension of the tumor, although growth based on bidimensional measurements (size measured as the product of the longest dimension of the tumor multiplied by its longest perpendicular measurement, the World Health Organization (WHO) criteria) was also calculated. Tumor growth rate was estimated using an exponential growth model. The week-4 screening visit scan and the baseline scan (3–5 days prior to start of protocol therapy) were used to estimate the growth rate prior to enrollment. The baseline scan and the first evaluation scan were used to determine the posttreatment growth rate.

Patients who were alive at 4 months without progression and had ≥30% increase in lung lesion tumor DT relative to pretreatment tumor growth rate were considered success. A rate of 30% of the subjects experiencing a “success” was considered encouraging and a rate of 10% as discouraging. This is similar to criteria established by Grignani et al. in a phase 2 trial of sorafenib for metastatic osteosarcoma [16]. However, success in the present trial is more stringent, as patients are required to be progression-free at 4 months and to have ≥30% increase in DT between the screening period and the subsequent evaluation period. Additionally, subjects whose untreated tumor growth rate was zero or very low (defined as a rate of increase that would not have progression within 4 months even if the tumor DT increased by <30%; “slow growth” cohort) were allowed into the study (up to 4 such cases) but were reported independently and not included in the primary analysis based on the “success” rate.

Simon's optimal two-stage design was employed, where initially 18 subjects were to be enrolled. If ≤2 subjects were classified as a “success,” the study would close. With “success” in ≥3 subjects, an additional 17 subjects would be accrued (total of 35), where ≥7 with “success” were required to determine the regimen worthy of further consideration in this population. The design provided 90% power for a positive finding if the true success rate was 30%, and a one-sided type I error of 5% for observing a promising result with a true success rate of 10%. Planned secondary endpoints were response rate (RR), OS, PFS, tumor growth rate, and PK effects. Survival analysis was conducted by using the Kaplan–Meier method (follow-up assessed by reverse Kaplan–Meier).

## 3. Results

### 3.1. Patients

The study enrolled 12 patients between October 10, 2013, and November 7, 2016, with follow-up continuing until January 10, 2018. The study was terminated prematurely prior to completion of the first stage due to withdrawal of financial support by the sponsor.

Patient characteristics are given in Table 1. The median age was 32 years with 58% female and 58% Caucasian. All patients had conventional osteosarcoma, and most patients' tumors were grade 3 (58%). There were 4 patients enrolled that had very low tumor growth rate prior to treatment. The median (95% CI) follow-up was 14.8 (range 7.2, 16.4) months. Drug discontinuation was due to progression of disease in 10 patients (83%); grade 3 ALT/AST begins on the 3rd cycle of treatment in 1 patient (8%), and grade 2 vomiting and grade 2 dyspnea (unrelated) is followed by rapid clinical deterioration due to pleural effusion in 1 patient (8%).

### 3.2. Efficacy

**Figure 1:**
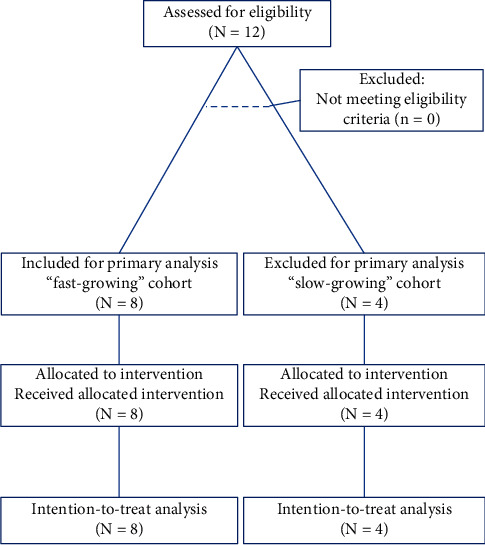
Consort diagram.

This study required two scans prior to treatment to enable assessment of tumor growth. As the study was closed with 8 patients in the primary cohort, as opposed to the planned 18 patients for the first interim analysis, and 4 patients were accrued in the separate “slow growth” cohort, we present the tumor growth for the patients in two figures.  Figure 1 shows the patient inclusion flowchart.

**Figure 2:**
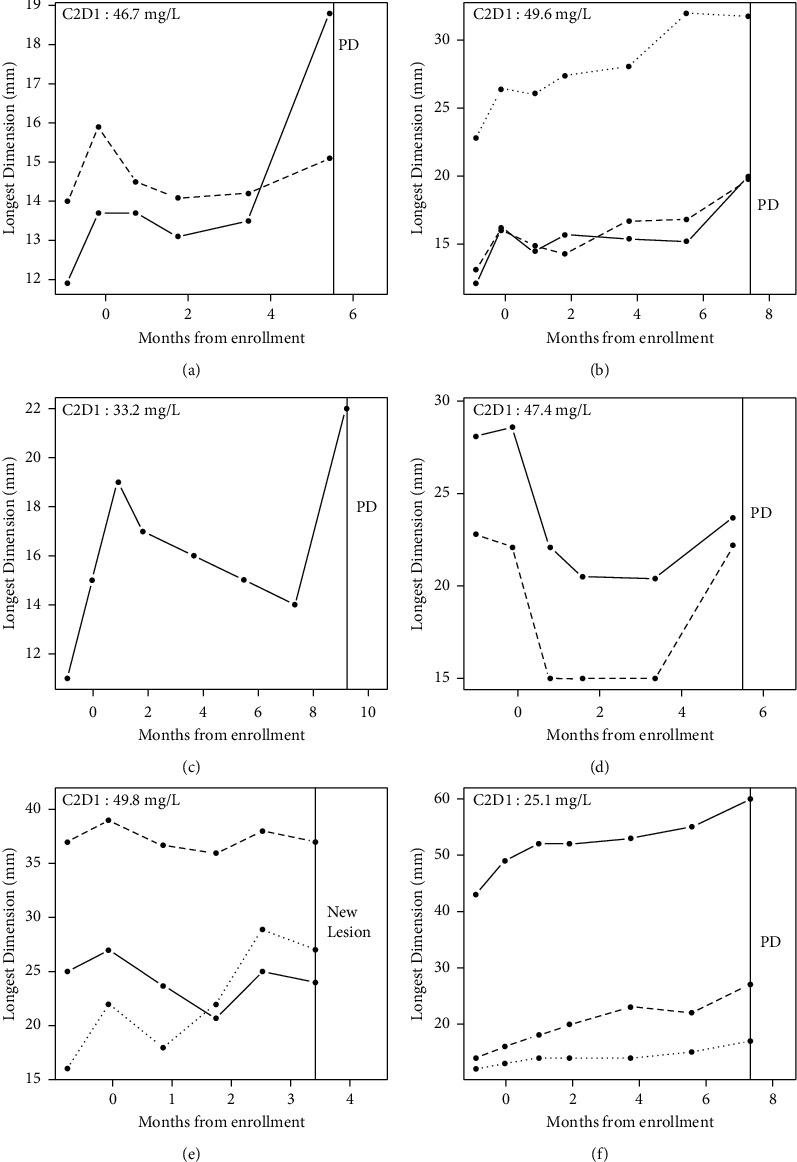
Growth rates in patients with clinical benefit with pazopanib.

In Figure 2, we present the 6 patients with evidence of benefit. Figures 2(a)–2(c) represent the 3 patients in the primary cohort that was a “success” by the preplanned criteria (progression-free at 4 months and a reduction in the tumor growth rate by ≥30%). This represents 3 of the 8 primary cohort patients (37.5%). This was sufficient for passing the first interim analysis, despite only 8 of the 18 of the first-stage planned accrual was reached in the primary cohort at time of study closure. Figure 2(d) shows a patient in the “slow growth” cohort who experienced a partial response. It is noteworthy that if considering the growth rate of the tumor in this patient by bidimensional measurement (WHO criteria) instead of the single longest dimension (RECIST v 1.1 criteria), the tumor would not have been considered “slow-growing,” rather it would have been included into the primary cohort (Supplemental Table 1). Figure 2(e) shows a patient who had a reduction in tumor growth in all three lesions, but a new lesion was noted prior to 4 months, and Figure 2(f) shows a patient progression-free for 7.3 months, but the change in growth rate, per our specified rules, did not achieve the 30% reduction.

**Figure 3:**
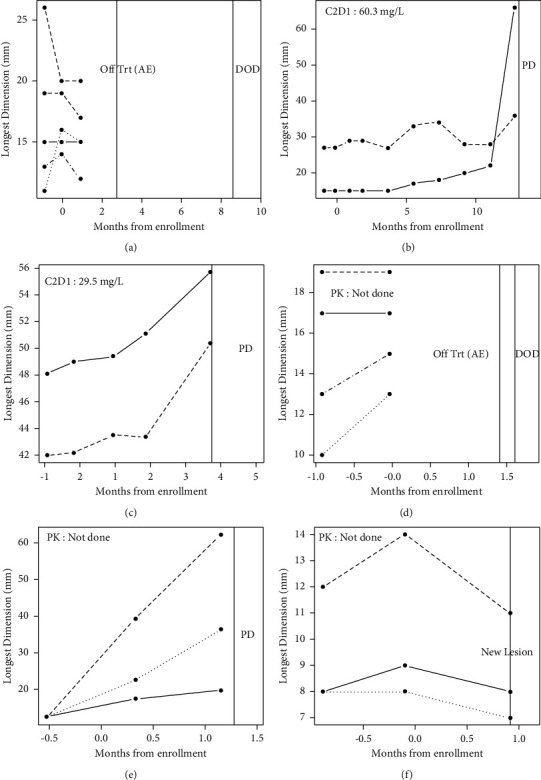
Growth rates in patients with absence of clinical benefit from pazopanib.

Figure 3 shows the 6 patients where the tumor growth patterns do not supply evidence of benefit. Figure 3(a) shows a patient in the “slow” growth rate cohort that stopped therapy rapidly due to an adverse event; Figure 3(b) shows a patient in the “slow growth” cohort who progressed after 13.1 months. Figure 3(c) shows the “slow growth” group that progressed prior to 4 months. Figure 3(d) shows the primary cohort but stopped therapy due to adverse events prior to the first on-treatment imaging. Figure 3(e) shows the primary cohort that showed no change in tumor growth. Figure 3(f) shows a patient with a decrease in tumor growth, but who revealed a new lesion at the first posttreatment imaging. The tumor growth rate data for both longest dimensional and bidimensional measurements are given in Supplementary Table 1. The best overall response data are given in Table 2.

### 3.3. Adverse Events

Grade 1-2 diarrhea was the most common TEAE (8 patients, 67%), followed by grade 1-2 nausea/vomiting (6 patients, 50e%), grade 1-2 hypothyroidism (4 patients, 33%), and grade 1 anorexia (4 patients, 33%). The following four grade 3 TEAEs occurred in 4 different patients: hypertension (1 patient, 8%), decrease in ejection fraction (1 patient, 8%), elevation in bilirubin level (1 patient, 8%), and elevated transaminases (1 patient, 8%). There were no grade 4 or 5 TEAEs (Table 3). Toxicity led to study discontinuation in 2 patients (17%). Grade 3-4 AEs considered unlikely related to treatment include one patient with a grade 4 attempted suicide, a patient with grade 4 alkaline phosphatase and grade 3 hyponatremia, and a patient with a grade 3 pulmonary embolism. There were no new previously undescribed TEAEs.

### 3.4. Pharmacokinetic Analysis

Pazopanib trough steady-state concentrations on day 1 of cycle 2 ranged from 25.3 to 60.3 mg/L (Supplemental Table 1). A correlation between clinical benefit and pazopanib trough steady-state concentrations was not demonstrable.

## 4. Discussion

In this trial, we evaluated the efficacy and toxicity of pazopanib, a multitargeted tyrosine kinase inhibitor, in patients with osteosarcoma with pulmonary metastases. There is a significant unmet need for new therapies in this population due to their poor prognosis and limited number of effective treatments.

This study closed prematurely due to withdrawal of funding by the sponsor. Consequently, the primary endpoints of PFS at 4 months concomitant with a demonstrated increase in tumor DT relative to the pretreatment growth rate were not fully assessable. Regardless, this study documented significant disease stability and a substantial reduction in tumor growth in 3 of the 8 primary cohort patients (37.5%). In fact, in each of those three patients, the tumors were reduced in size after initiation of pazopanib, but did not meet criteria for response. Accordingly, despite the accrual of only 12 patients (8 in primary cohort and 4 in “slow growth” cohort), the study would have successfully passed the first stage of Simon's optimal two-stage design to proceed to the second stage. Furthermore, a partial response was documented in a patient in the “slow growth” cohort. The 6 patients who did not have evidence of drug activity included 2 patients who stopped treatment at or before the first evaluation due to AEs: a patient with tumor reduction at the first evaluation but new lesions and one patient with a very fast-growing tumor.

In a nonrandomized trial, we acknowledge disease stability could be attributed to tumor growth variability rather than drug activity. However, the growth pattern pre- and post-treatment initiation observed for each patient provides substantial evidence supporting the activity of pazopanib. This is particularly meaningful for an aggressive disease such as osteosarcoma. Moreover, we propose that tumor growth rate is a relevant and meaningful surrogate endpoint in evaluating activity of new therapeutic agents in phase 2 trials. This is especially true for antiangiogenic agents such as pazopanib; these are cytostatic agents that delay tumor growth without necessarily decreasing tumor size. Furthermore, the objective response rate for cytotoxic chemotherapy like gemcitabine and docetaxel for relapsed osteosarcoma is low (7–13%), perhaps related to malignant osteoid matrix that often fails to regress radiographically despite cytocidal activity [17, 18]. Of note, our study intended to include children >16 years; however, in final analysis, all the patients were adults (age 18–72). Thus, the efficacy and toxicity profile of pazopanib in children could not be assessed. PK data were also too limited to provide a clear signal, although day 1 cycle 2 concentration results are presented (Figures 2 and 3).

In general, pazopanib was relatively well-tolerated. There were no grade 4 or 5 TEAEs. Grade 3 TEAEs occurred in 4 patients but required drug discontinuation in only 1 patient. Most of the observed TEAE's were grades 1 and 2. The most common TEAEs were diarrhea, nausea/vomiting, hypothyroidism, and anorexia. All of the TEAEs observed have been previously reported for pazopanib [13–15]. Drug discontinuation due to TEAEs occurred in 17% of patients (2 patients).

A correlation between clinical benefit and pazopanib trough steady-state concentrations was not demonstrated in this study. Clinical activity has been reported to be lower in patients with trough steady-state concentrations <20.5 mg/L [19, 20]. In the cohort with evidence of benefit, concentrations ranged from 25.1 to 49.8 mg/L (Figure 2), consistent with prior reports. PK data were not available for 4 subjects in the cohort where tumor growth patterns do not supply evidence of benefit (Figures 3(a), 3(d)–3(f)). Regardless, for the 2 subjects where PK data were available (Figure 3), both were >20.5 mg/L, including the highest trough steady-state concentration (60.3 mg/L) documented (Figure 3(b)). The absence of correlation likely is a result of the small number of subjects overall and insufficient PK data in the cohort without evidence of benefit.

This multicenter phase 2 study demonstrated that pazopanib can reduce the growth rate of pulmonary metastases that develop in patients with metastatic osteosarcoma. While a confirmed response was not observed in the primary analysis cohort (a partial response was observed in the slow growth cohort), the results provide rationale for further study on mechanisms of drug resistance and combining pazopanib with other cytotoxic therapies. This is consistent with the approach for other multitargeted tyrosine kinase inhibitors such as lenvatinib in combination with ifosfamide and etoposide [21]. We believe further that the presentation of individual tumor growth patterns provide significant added insight that would be missed if reporting only on response and PFS.

## 5. Conclusion

The primary end points of 4-month PFS together with increase in tumor DT ≥30% could not be fully evaluated in the current trial, as the study was prematurely terminated due to withdrawal of funding by the sponsor. Regardless, this study still demonstrated notable antitumor activity of pazopanib in recurrent osteosarcoma metastatic to the lung with disease stability and a substantial reduction in tumor growth in 3 of the 8 primary cohort of patients (37.5%). Furthermore, a partial response was documented in 1 of the 4 patients in the “slow growth” cohort. A larger trial of pazopanib that includes a broader age demographic (i.e., ≥13 years) should be considered, as a significant population of patients with osteosarcoma would typically be children and adolescents. In the interim, pazopanib can be considered an option for adult patients who fail to respond to chemotherapy.

## Figures and Tables

**Table 1:** Patient characteristics.

Number of patients treated	12
Median age (range) years	32 (18–72)
Median BMI (range)	30.18 (18.77–48.28)
Gender	
Female	7 (58%)
Male	5 (42%)
Race	
African American	1 (8%)
Asian	1 (8%)
Caucasian	7 (58%)
Hispanic	2 (17%)
Unknown	1 (8%)
Performance status (ECOG) at cycle 1, day 1^a^	
0	4 (33%)
1	5 (42%)
2	2 (17%)
Stage at consent	
IVA	6 (50%)
IVB	6 (50%)
Grade at consent^b^	
1	1 (8%)
2	0 (0%)
3	2 (17%)
4	7 (58%)

^a^Missing for one patient at C1D1 (at screening ECOG 0). At screening, ECOG 0/1 was 4/8. ^b^Missing for two patients.

**Table 2:** Best overall response.

Primary analysis group	
SD > 4 months	4
PD	3
N/A	1 (toxicity)

Subgroup of patients with low tumor growth rate	
PR	1
SD > 4 months	1
PD	1
N/A	1

**Table 3:** All treatment-related toxicities (possibly, probably, or definitely related)^a^.

Adverse event	Treatment arm		
	Pazopanib 600 mg once daily for a 28-day cycle (*n* = 12)		
	Grade 1	Grade 2	Grade 3
Hypertension	1 (8%)	1 (8%)	1 (8%)
Ejection fraction decreased	—	1 (8%)	1 (8%)
Elevated bilirubin	—	—	1 (8%)
Transaminases	—	—	1 (8%)
Fatigue	1 (8%)	2 (17%)	—
Pain	1 (8%)	2 (17%)	—
Diarrhea	7 (58%)	1 (8%)	—
Nausea/vomiting	5 (42%)	1 (8%)	—
Hypothyroidism	3 (25%)	1 (8%)	—
Neutropenia	2 (17%)	1 (8%)	—
Anemia	—	1 (8%)	—
Body aches	—	1 (8%)	—
Palmar-plantar erythrodysesthesia	—	1 (8%)	—
Right mandibular infection	—	1 (8%)	—
Tachycardia	—	1 (8%)	—
White blood cell count decreased	—	1 (8%)	—
White blood cell count increased	—	1 (8%)	—
Anorexia	4 (33%)	—	—
Abdominal pain	3 (25%)	—	—
Platelet count decreased	3 (25%)	—	—
Dizziness	2 (17%)	—	—
Weight loss	2 (17%)	—	—

^a^All treatment-related (possibly, probably, or definite) except for grade 1 AEs that occurred in only one patient.

## Data Availability

The raw, unanalyzed data are available upon request to Dr. Warren Chow, as an Excel file.

## Supplementary Materials

Supplementary Materials Supplementaary Table 1: numerical growth rates^a^. Column (1) figure number. (2) Baseline doubling time according to longest dimensions for individual target lesion(s) based on two measurements prior to start of treatment cycle 1. (3) Baseline doubling time according to 2-dimensional (D) area for individual target lesion(s) based on two measurements prior to start of treatment cycle 1. (4) Baseline doubling time for sum of the longest dimensions for ALL of the tumors. (5) Baseline doubling time for sum of the 2D area for all of the tumors. (6) Classification of subject as fast growing cohort (Y/N). (7) Doubling time on treatment based on measurement pretreatment and next measurement on-treatment. (8) Treatment success according to predefined statistical analysis (Y/N). (9) Pazopanib trough steady-state concentration on day 1 of cycle 2 prior to its administration (mg/L). Row (1) longest dimension; row (2) 2D area.
