# Supplementary material for: Pazopanib in Patients with Osteosarcoma Metastatic to the Lung: Phase 2 Study Results and the Lessons for Tumor Measurement
Source: J Oncol. 2022 Jan 15;2022:3691025. doi: 10.1155/2022/3691025 (PMC8783732; doi:10.1155/2022/3691025)
Supplement: Supplementary Materials — Supplementaary Table 1: numerical growth ratesa. Column (1) figure number. (2) Baseline doubling time according to longest dimensions for individual target lesion(s) based on two measurements prior to start of treatment cycle 1. (3) Baseline doubling time according to 2-dimensional (D) area for individual target lesion(s) based on two measurements prior to start of treatment cycle 1. (4) Baseline doubling time for sum of the longest dimensions for ALL of the tumors. (5) Baseline doubling time for sum of the 2D area for all of the tumors. (6) Classification of subject as fast growing cohort (Y/N). (7) Doubling time on treatment based on measurement pretreatment and next measurement on-treatment. (8) Treatment success according to predefined statistical analysis (Y/N). (9) Pazopanib trough steady-state concentration on day 1 of cycle 2 prior to its administration (mg/L). Row (1) longest dimension; row (2) 2D area. [file 3691025.f1.docx]

**Supplemental Table 1. Numerical growth rates^a^**

**Col. 1 2 3 4 5 6 7 8 9**

| Figure | Doubling Time Baseline, LD  (Months) | Doubling Time  Baseline 2D (Area)  (Months) | DT_B  (Sum LD) | DT_B  (Sum 2D) | Fast  Sum (LD) | DT on Treatment  Row 1: LD  Row 2: Area (2D) | Success | PK mg/L |
| --- | --- | --- | --- | --- | --- | --- | --- | --- |
| 2A | 3.7,4.1  Med: 3.9 | 4.0,2.3  Med: 3.1 | 3.9 | 2.9 | Y | FL,-6.7  -70,-3.5 | **Y**  **SD 5.5** | 46.7 |
| 2B | 1.8,2.6,3.6  Med: 2.6 | 1.7,1.8,1.5  Med: 1.7 | 2.6 | 1.6 | Y | -6.4,-9.9,-61.8  -2.7,-36.1,-214.4 | **Y**  **SD 7.4** | 49.6 |
| 2C | 2.0  Med: 2.0 | 0.9  Med: 0.9 | 2.0 | 0.9 | Y | 2.8  1.3  Decrease size post 2^nd^ cycle | **Y**  **SD 9.2** | 33.2 |
| 2F | 4.5,4.4,7.4  Med: 4.5 | 1.7,3.4,3.3  Med: 3.3 | 4.8 | 3.1 | Y | 11.9,6.0,9.5  6.7,13.3,-33.3 | UNK  SD 7.3 | 25.1 |
| 2E | 6.2,9.1,1.5  Med: 6.2 | 3.3,-40.2,0.9  Med: 0.9 | 4.0 | 3.0 | Y | -4.9,-10.5,-3.2  -4.9,-1.4,-1.0 | N  NL 3.4 | 49.8 |
| 3F | 4.6,3.5, FL  Med: 4.6 | 2.2,1.3,3.5  Med: 2.2 | 5.4 | 1.7 | Y | -6.0, -2.9,-5.3  -2.8,-1.4,-5.3 | N  NL 1 | -- |
| 3E^b^ | 1.8,0.5,1.0  Med:1.0 | 1.1,0.3,0.5  Med: 0.5 | 0.8 | 0.4 | Y | 4.8,1.2,1.2  3.4,0.6,0.6 | N  PD 1.3 | -- |
| 3D | FL,FL,2.3,4.3  Med: Undef | -8.9,10.1,1.2,1.1  Med: 1.2 | 7.6 | 3.2 | Y | Adverse Event | N  DOD/PD^c^ 1.6 | -- |
| 2D | 34.9,-19.7  Med: 7.6 | 16.7, 3.6  Med: 10.2 | -156.2 | 7.5 | N | -2.5, -1.6  -0.9, -1.1 | **Y**  **PR 5.5** | 47.4 |
| 3A | FL,-2.3,1.6,8, FL  Med: Undef | 7.4,-3.8,0.9,3.5,4.7  Med: 2.2 | FL | 5.8 | N | FL,FL,-10.2,-4.3,-5.9  -8.3,-6.3,-2.3,-1.9,-3.8 | UNK  DOD/PD^c^ 8.6 | -- |
| 3C | 28.3,110.3  Med: 69.3 | -71.1,111.0  Med: 20.0 | 43.2 | -199.3  (FL) | N | 95.2,25.5  15.7,10.4 | UNK  PD 3.75 | 29.5 |
| 3B | FL, FL  Med: FL | 5.2, 9.6  Med: 7.4 | FL | 8.0 | N | FL, 10.2  FL,51.0 | UNK  SD 13.1 | 60.3 |

^a^ 13 of the 133 measurements after the first baseline had the longest dimension shorter than the perpendicular, likely related to whether the original orientation was maintained for the dimensions. For consistency, we used the longest dimension reported for each lesion even if recorded as the perpendicular measurement. Slow was determined by doubling time of 10.8 months or longer based on sum of LD (growth rate of 6.1% or less during 28 days). FL=Flat. NL=New Lesion.

^b^ Missing value for target right lung lesion #1 at first evaluation and this lesion was omitted.

^c^ Patient died without progressive disease.
